# Test data from pullout experiments on vetiver grass (*Vetiveria zizanioides*) grown in semi-arid climate

**DOI:** 10.1016/j.dib.2018.01.048

**Published:** 2018-01-31

**Authors:** Slobodan B. Mickovski, L.P.H. van Beek

**Affiliations:** a School of Engineering and Built Environment, Glasgow Caledonian University, United Kingdom; b Geosciences Department, University of Utrecht, The Netherlands

## Abstract

The data set presented in this article includes the results of pullout tests carried out on vetiver grass (*Vetiveria zizanioides*) growing on an abandoned terrace slopes in Spain. The results comprise tables showing the resistance of each tested vetiver plant to pullout forces applied to it at various angles. The dataset also contains the measurements of the displacement at each pullout force increment. The dataset also includes the plots of the pullout resistance of each vetiver plant against the measured displacement.

**Specifications Table**

**Table:** 

Subject area	*Biology, Engineering*
More specific subject area	*Ecological engineering*
Type of data	*Tables and graphs*
How data was acquired	*in situ measurement of the force and displacement during pullout. The force was measured using a hand-held portable force gauge (Alluris FMI-100)*
Data format	*Raw data presented in tables and analyzed on graphs*
Experimental factors	*Before each pullout test the soil surface in a radius 30 cm around the plant was carefully cleared from the litter, exposing the stem base*
Experimental features	*A strong PVC rope (3 mm diameter) padded with soft tissue in order not to destroy the plant material was then tied around the stem base of the plant. The other end of the rope was connected to a hand-held portable force gauge for accurate measurement of uprooting force. In order to mimic the forces applied to the plant during runoff and sediment impoundment, the pullout force was applied parallel to the slope in downslope direction. The force was applied manually with a rate of 10 mm per minute, recording the change in resistance along the way. The test was terminated once the resisting force dropped sharply and the plant was uprooted.*
Data source location	*Almudaina, Spain (X= 729275 Y= 4293850 and Z= 480 m on UTM 30 s)*
Data accessibility	*The data is with this article.*

**Value of the data**

• This data set provides an insight into the behavior of vetiver grass grown in semi-arid climate during potential erosion or shallow landslide event

• This data set can be used to develop a reinforcement models for vegetated soils
• This data set can be combined with published data on root distribution of vetiver to derive the strength that vetiver root systems can impart on soil
• This data set can be combined with published data on above-ground vetiver plant morphology to derive the sediment retention capacity of vetiver plants
• This dataset can serve as a benchmark for measurement and presentation of parameters required for derivation of root reinforcement models and soil-root interaction

## 1 Data

**Fig. 1:**
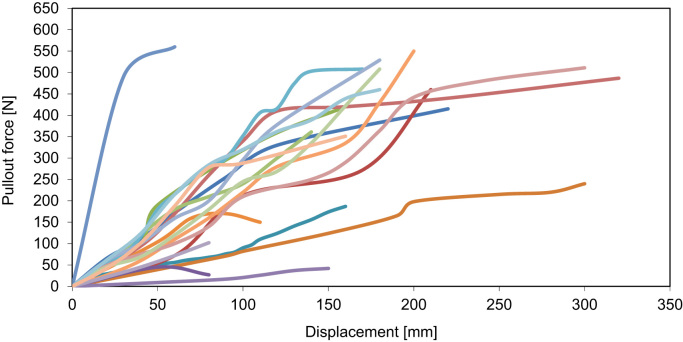
Pullout resistance of each vetiver plant against the measured displacement.

The data set of the results of pullout tests carried out on vetiver grass (*Vetiveria zizanioides*) growing on row on an abandoned terrace slopes in Spain is shown on Table 1, Table 2, Table 3. The results show the resistance of each tested vetiver plant to pullout forces applied to it. This dataset also contains the measurements of the displacement at each pullout force increment. Fig. 1 shows the plots of the pullout resistance of each vetiver plant against the measured displacement.

**Table 1:** Pullout resistance of vetiver plants from Row A.

***Sample:***	***A2***		***Sample:***	***A3***		***Sample:***	***A4A***		***Sample:***	***A12***		***Sample:***	***A13***
Direction of pull [°]:	45		Direction of pull [°]:	60		Direction of pull [°]:	60		Direction of pull [°]:	60		Direction of pull [°]:	60
Diam. At base [mm]	68			30			35			56			69
Displacement	Force		Displacement	Force		Displacement	Force		Displacement	Force		Displacement	Force
[mm]	[N]		[mm]	[N]		[mm]	[N]		[mm]	[N]		[mm]	[N]
0	0.0		0	0.0		0	0.0		0	0.0		0	0.0
20	68.0		20	134.0		20	132.0		20	68.0		20	80.0
40	118.0		40	190.0		40	200.0		40	133.0		40	122.0
60	200.0		60	216.0		60	260.0		60	186.0		60	188.0
80	247.0		80	290.0					80	285.0		80	235.0
120	407.0		120	370.0					120	365.0		100	301.0
160	477.0		140	389.0					160	484.0		120	379.0
180	540.0		200	506.0					200	615.0		140	399.0
												160	478.0
												200	562.0

**Table 2:** Pullout resistance of vetiver plants from Row B.

***Sample:***	***B1***	***Sample:***	***B2A***	***Sample:***	***B2B***	***Sample:***	***B3A***	***Sample:***	***B4A***	***Sample:***	***B4B***	***Sample:***	***B5***	***Sample:***	***B6***	***Sample:***	***B7A***	***Sample:***	***B7B***	***Sample:***	***B8***
Direction of pull [°]:	18	Direction of pull [°]:	0	Direction of pull [°]:	14	Direction of pull [°]:	0	Direction of pull [°]:	0	Direction of pull [°]:	0	Direction of pull [°]:	0	Direction of pull [°]:	0	Direction of pull [°]:	0	Direction of pull [°]:	0	Direction of pull [°]:	0
Diam. At base [mm]	61		57		57		80		73		73		59		90		56		56		30
Displac.	Force	Displac.	Force	Displac.	Force	Displac.	Force	Displac.	Force	Displac.	Force	Displac.	Force	Displac.	Force	Displac.	Force	Displac.	Force	Displac.	Force
[mm]	[N]	[mm]	[N]	[mm]	[N]	[mm]	[N]	[mm]	[N]	[mm]	[N]	[mm]	[N]	[mm]	[N]	[mm]	[N]	[mm]	[N]	[mm]	[N]
0	0.0	0	0.0	0	0.0	0	0.0	0	0.0	0	0.0	0	0.0	0	0.0	0	0.0	0	0.0	0	0.0
40	94.0	20	51.0	50	9.0	10	30.0	20	26.0	20	60.0	20	48.0	20	45.0	20	22.0	20	61.0	20	42.0
60	170.0	40	120.0	90	17.0	20	56.0	40	60.0	40	100.0	40	75.0	40	69.0	40	45.0	40	123.0	40	101.0
80	260.0	60	180.0	110	26.0	30	74.0	60	110.0	60	159.0	60	100.0	60	120.0	60	72.0	60	215.0	60	188.0
100	340.0	80	210.0	130	37.0	40	116.0	80	160.0	80	200.0	80	140.0	80	180.0	80	102.0	80	284.0	80	278.0
120	410.0	100	240.0	150	42.0	50	180.0	100	220.0	100	296.0	100	210.0	100	245.0			100	320.0	100	288.0
160	420.0	120	300.0			60	215.0	120	280.0	120	378.0	140	250.0	120	269.0			120	362.0	160	351.0
220	440.0	140	361.0			70	250.0	160	335.0	180	529.0	160	290.0	140	335.0			140	389.0		
320	486.7					80	278.0	180	430.0			180	364.0	160	422.0			160	439.0		
							90	299.0	200	550.0		200	442.0	180	508.0			180	460.0		
							100	352.0					240	480.0							
							110	406.0					300	511.0							
							120	416.0													
							130	474.0													
							140	503.0													
							170	508.0

**Table 3:** Pullout resistance of vetiver plants from Row D.

***Sample:***	***D6***	***Sample:***	***D5-I***	***Sample:***	***D5B***	***Sample:***	***D5C***	***Sample:***	***D4***	***Sample:***	***D3***	***Sample:***	***D1A***	***Sample:***	***D1B***
Direction of pull [°]:	0	Direction of pull [°]:	0	Direction of pull [°]:	0	Direction of pull [°]:	0	Direction of pull [°]:	0	Direction of pull [°]:	0	Direction of pull [°]:	0	Direction of pull [°]:	50
Diam. at base [mm]	50		91		91		91		92		35		76		76
Displac.	Force	Displac.	Force	Displac.	Force	Displac.	Force	Displac.	Force	Displac.	Force	Displac.	Force	Displac.	Force
[mm]	[N]	[mm]	[N]	[mm]	[N]	[mm]	[N]	[mm]	[N]	[mm]	[N]	[mm]	[N]	[mm]	[N]
0	0.0	0	0.0	0	0	0	0	0	0.0	0	0.0	0	0.0	0	0.0
10	21.7	90	71.0	20	65	60	72	40	100.0	50	45.0	50	104.0	30	490
15	26.0	100	82.0	40	110	100	212	50	190.0	80	27.0	70	160.0	60	560
20	29.0	150	124.0	60	172	170	272	110	340.0			90	170.0		
25	33.0	190	165.0	90	255	210	460	160	420.0			110	150.0		
30	33.0	200	198.0	120	330										
35	38.0	250	215.0	220	415										
40	43.0	280	220.0												
45	48.0	300	240.0												
50	51.0														
55	54.0														
60	56.0														
65	60.0														
70	63.0														
75	65.0														
80	68.0														
85	71.0														
90	76.0														
95	80.0														
100	90.0														
105	97.0														
110	110.0														
120	124.0														
130	141.0														
140	156.0														
150	174.0														
160	187.0														

## 2 Experimental design, materials and methods

In order to investigate the pullout resistance of vetiver grass, 24 plants were randomly chosen from a plantation on an abandoned terrace near Almudaina, Spain and were used as a test sample [1]. Before each pullout test the soil surface in a radius 30 cm around the plant was carefully cleared from the litter, exposing the plant stem base. A strong PVC rope (3 mm diameter) padded with soft tissue in order not to destroy the plant material was then tied around the stem base of each plant. The other end of the rope was connected to a hand-held portable force gauge (Alluris FMI-100) for accurate measurement of the uprooting force. In order to mimic the forces applied to the plant during runoff and sediment impoundment, as well as shallow landslides, the pullout force was applied at different angles to the slope in downslope direction. The force was applied manually with a rate of 10 mm per minute, recording the change in resistance along the way. The displacement of the plant during the pullout was measured using a tape measure with a 1 mm precision (Table 1, Table 2, Table 3). The test was terminated once the resisting force dropped sharply and the plant was uprooted. The data on pullout resistance was then plotted against plant displacement (Fig. 1) to show the plant behavior during the process.
